# Statins and Postoperative Infections: A Randomized Clinical Trial

**DOI:** 10.5812/jjm.9456

**Published:** 2014-04-01

**Authors:** Shervin Shokouhi, Ilad Alavi Darazam, Giv Sharifi, Latif Gachkar, Anahita Amirsardari, Mohammad Samadian

**Affiliations:** 1 Department of Infectious Disease, Loghman Hospital, Shahid Beheshti University of Medical Sciences, Tehran, IR Iran; 2 National Research Institute of Tuberculosis and Lung Diseases, Shahid Beheshti University of Medical Sciences, Tehran, IR Iran; 3 Department of Neurosurgery, Loghman Hospital, Shahid Beheshti University of Medical Sciences, Tehran, IR Iran; 4 Infectious Diseases and Tropical Medicine Research Center, Shahid Beheshti University of Medical Sciences, Tehran, IR Iran

**Keywords:** Statin, Post-Operative Period, Infection, Neurosurgery

## Abstract

**Background:**

Observational studies, rather than randomized trials, revealed that statins might be associated with other benefits.

**Objectives:**

The present study aimed at evaluating the preventive effects of lovastatin when used as a prophylactic agent for early and late infective complications after surgery.

**Patients and Methods:**

A total of 149 patients undergoing elective intracranial and spinal surgeries, were enrolled in a double- blind randomized clinical trial in the department of neurosurgery of a teaching hospital. An amount of 20 mg lovastatin and the same dose of placebo, one day before the operation and three days after the surgery, were used for cases and controls, respectively. The patients were evaluated for local and systemic infections during hospitalization and 10, 30, 60 and 90 days after discharge.

**Results:**

A total of 149 patients, 78 men and 71 women with a mean age of 40.3 ± 16.5, were assigned to prophylactic protocols. 46 and 103 patients were in the case and control groups, respectively. Eight episodes of infection were detected, including six bacterial meningitis and two episodes of hospital- acquired pneumonia. All of the patients with documented postoperative infections were part of the placebo group, however, there were no significant statistical differences between the groups (P = 0.059).

**Conclusions:**

In spite of the differences between the two groups, the results did not significantly support the preventive effect of statins in postoperative infections.

## 1. Background

Despite recent efforts to prevent surgical site infections (SSIs), these infections are not infrequent. According to data retrieved from the National Center For Health Statistics (1) and the National Healthcare Safety Network (NHSN) (2), up to 1 million SSIs occur annually in the United States and the burden results 3.7 million excess hospital days and 1.6 billion dollars in excess costs (3). SSI leads to more intensive care unit admissions and also more complications after discharge. In addition to SSI, the patients, after surgery, are predisposed to other localized and systemic infections, including hospital- acquired pneumonia, urinary tract infection, and catheter- associated infection, etc. 3-hydroxy-3-methylglutaryl coenzyme A reductase inhibitors, so called statins, are involved in the regulation of cholesterol synthesis. The role of statins has been demonstrated in the treatment of dyslipidemia and reducing the risk of coronary artery disease (4). Statins also deplete nonsterol cholesterol precursors, the isoprenoids, necessary for prenylation of critical membrane proteins that regulate cellular communication, including inflammatory response (4).

Observational studies, rather than randomized trials, revealed that statins might be associated with other benefits. Decreasing the risk of dementia, protecting against the development of lung cancer (5), preserving renal function (6), and having protective effects against infection and sepsis (7) are known as pleiotropic effects of statins (8, 9). As such, there is a potential confounding and bias that patients receiving better medical care, and those more adherent to medical therapy, may be more likely to be taking statins and are also at lower risk for certain noncardiovascular diseases (9). Also some investigators have suggested that the protective associations reported in many of these studies ,could reflect bias from “healthy user” effects; that is, statin users tend to have less severe comorbidity and better functional status than nonusers and are more likely to practice other healthy behaviors (10).

## 2. Objectives

The present study aimed at evaluating the preventive benefits of lovastatin when used as a prophylactic agent for early and late infective complications after neurosurgical procedures.

## 3. Patients and Methods

Consecutive 149 patients, (sampling with the power of 0.95 and the significance level of 0.05), undergoing elective intracranial and spinal surgeries, were enrolled in a double- blind randomized clinical trial in the department of neurosurgery of a teaching hospital. The size of the control group was approximately twice that of the case group. An amount of 20 mg lovastatin and the same dose of placebo, one day before the operation and three days after the surgery, were used for cases and controls, respectively. Also, all patients received vancomycin and ceftriaxone before, during and 48 hours after the surgery based on the Prophylactic strategy of this department.

All of the patients with suggestive symptoms of infection prior to the procedure, immunocompromised individuals and those previously on statins treatment, were excluded. The patients were evaluated for local and systemic infections during hospitalization and 10, 30, 60 and 90 days after their discharge. Surgical site infection included local induration, purulent discharge, serous discharge with positive culture, superficial and deep subcutaneous abscesses with or without positive cultures, debilitating pain in vertebral and spinal surgeries, fever more than 38.5˚C, bacteremic episodes (two repeated positive cultures of one microorganism), one positive blood culture with undetermined fever, positive culture of responsible microorganism in distant location, magnetic resonance imaging and computed tomography findings suggestive of infection, bone flap osteitis, epidural and subdural empyema, brain abscess, meningitis and systemic infection including pneumonia. 

All patients were evaluated by an infectious disease specialist, neurosurgeon and radiologist. Fisher exact test was used for evaluating the lovastatin effect on postoperative infection rate. Influencing factors on postoperative infection were analyzed with multivariate logistic regression analysis. Level of significance was P value < 0.05. The Committee of Medical and Research Ethics of Shahid Beheshti University of Medical Sciences approved the ethical issues of this clinical trial.

## 4. Results

A total of 149 patients, 78 men and 71 women with a mean age of 40.3 ± 16.5, were assigned to prophylactic protocols. Forty-six and 103 patients were in the case and control groups, respectively (Table 1). Eight episodes of infections were seen, including six bacterial meningitis and two cases of hospital- acquired pneumonia. All of the patients with documented postoperative infection were placed in the placebo group however, there were no significant statistical differences between the groups (P = 0.059). All of the infections were documented during the hospitalization, but no episodes were seen after discharge. According to the criteria for skin and soft tissue infections (see the materials and methods), there was no documented infection.

**Table 1.** Demographic and Characteristics of Patients ^a^, ^b^

	Results
**Age**	40.30 ± 16.536
**Gender**	
Male	78 (52.3)
Female	71 (47.7)
**Lovastatin prophylaxis**	
Yes	46 (30.9)
No	103 (69.1)
**Postoperative infection**	
Yes	8 (5.4)
No	141 (94.6)
**No ICU admission**	
Not admitted	71 (47.7)
**ICU admission, d**	
1-5	70 (47.0)
5-10	6 (4.0)
> 10	2 (1.3)
**Surgical location**	
Spinal	73 (49.0)
Brain	76 (51.0)
**Duration of surgery, h**	
< 4	76 (51.0)
4-8	68 (45.6)
> 8	5 (3.4)
**Opened dura mater during the operation**	
Yes	63 (42.3)
No	86 (57.7)
**History of diabetes**	
Yes	9 (6.0)
No	140 (94.0)
**History of hypertension**	
Yes	12 (8.1)
No	137 (91.9)
**History of COPD**	
Yes	4 (2.7)
No	145 (97.3)
**History of CHF**	
Yes	5 (3.4)
No	144 (96.6)
**History of corticosteroid**	
Yes	4 (2.7)
No	145 (97.3)
**Underlying pathology**	
Tumoral	63 (42.3)
Nontumoral	86 (57.7)

^a^ Abbreviations: CHF, congestive heart failure; COPD, chronic obstructive pulmonary disease; ICU, intensive care unit.

^b^ Data are presented as No. (%) or Mean ± SD.

Intracranial procedures resulted more infective complications than surgeries on the spinal canal (P = 0.006). All of the postoperative infections occurring in 86 surgical procedures, needed opening of the dura during operation compared with surgeries with intact dura (P = .013). Underlying diseases including diabetes mellitus, chronic obstructive pulmonary disease, hypertension and congestive heart failure did not increase postoperative infection rate in both groups. Gender of the patients, duration of operation and device implantation in the surgical site, previous history of surgical site infection, recent history of corticosteroids, underlying histopathology and smoking history were unremarkable factors in postoperative infections (P > 0.05). The patients with infection were younger than the others (P = 0.014). Postoperative infections led to longer hospitalization (more than five days) and more intensive care unit admissions (P = 0.007).

## 5. Discussion

Studies on the effect of statins in improving mortality and morbidity of hospitalized patients are divided into studies evaluating either direct lipid lowering and cardio- or cerebrovascular protection effects or minimizing other inflammatory complications, including renal failure, bacteremia, infective events, etc (5-7). In a multicenter, randomized, double- blind, and prospective trial, atorvastatin had no statistically significant effect on the composite primary end point of cardiovascular death, nonfatal myocardial infarction, and stroke in patients with diabetes receiving hemodialysis. Fatal infections were also not different in both groups (10). As mentioned above, other studies focused on other pleiotropic effects of statins particularly fatal infections in hospitalized or intensive care units. Pneumonia and sepsis are frequently assessed by studies evaluating the prophylactic effects of statins.

Despite the beneficial effects of statins in the management of infections, published in observational studies, new randomized trials revealed no additional benefits. The differences are mainly due to structural design of studies that lead to selection bias and biased estimation of the effects despite extensive efforts of investigators to decrease confounding factors. In a cohort study of 112 patients with ischemic stroke assessing the impact of early statin use on the risk of poststroke infection, data suggested that early statin use appears to be associated with and increases the risk of poststroke infection. It was concluded that this risk might be attributable to increases in plasma IL-1ra (11). On the other hand, in evaluating the safety and tolerability of atorvastatin in the Collaborative Atorvastatin Diabetes Study (CARDS), the infection rate eventually became similar in both groups (with and without atorvastatin) (12). 

In a retrospective review of 388 bacteremic infections due to aerobic Gram-negative bacilli and *Staphylococcus aureus*, there was a significant reduction in both overall (6% vs. 28%) and attributable (3% vs. 20%) mortality among patients taking statins compared with patients not taking statins. Among the statin group, diabetes, hypertension, and coronary artery disease were more prevalent, and there were more skin and soft tissue infections identified as sources of bacteremia. These data suggest a potential clinical role of statins in bacteremic infection; however, the mechanism by which mortality was reduced, remained undefined (4). A recent systematic review and meta-analysis evaluated sixteen cohorts addressing the role of statins in treating infections and infection prevention (nine and seven cohorts, respectively). 

Patients in the mentioned cohorts were different based on their underlying diseases and settings including vascular diseases, chronic kidney diseases, diabetes, intensive care unit-acquired infections, and in general practice (13). Their evaluation revealed no evidence to support the hypothesis that statins decrease the risk of infection with a relative risk of 1.00 for infection and 0.97 for infection- related mortality. So it did not support the causal protective effect of statins as reported by observational studies. The mentioned cohorts did not include postoperative infection prophylaxis, and overall studies on prophylactic effects of statin, specifically on postoperative infections, are very rare. 

In a cohort evaluation of patients undergoing coronary artery bypass graft (CABG) and/or valve surgery in which 1248 received a statin preoperatively and 686 did not, a significant reduction was shown in the development of infection among patients receiving statins, based on logistic regression. Despite the limitations of this nonrandomized trial, the investigators concluded that preoperative statin use is associated with a reduction in patients' odds of developing a postoperative infection following cardiac surgery (14). Furthermore, another study investigated the relationship between statins and postoperative wound complications. It showed that statin use did not affect the risk of wound infection or delayed wound healing. 

Statin use was, however, associated with an increased rate of local postoperative bleeding complications (P = 0.01). Patients who had undergone inguinal herniorrhaphy while on statins had an increased risk of postoperative wound hematoma/hemorrhage (15). As mentioned above, effect of statins is still debated; the argument around the preventive effect of statins in postoperative infections remains due to heterogeneity and limitations in the number of studies and lack of randomized clinical trials. Our study is the only randomized clinical trial established and presented in this way in the published literatures. Despite the differences between the two groups, this study did not significantly support the preventive effects of statins in postoperative infections. However, limitations may be due to a small number of subjects on statin and short duration of prophylactic intake of lovastatin. To demonstrate the true causal protective effect of statins, it is recommended to plan a multi-centric controlled trial with large number of subjects, multiple doses and duration.
